# Multidetector Computed Tomography Angiography Findings of Chronic-Contained Thoracoabdominal Aortic Aneurysm Rupture with Severe Thoracal Vertebral Body Erosion

**DOI:** 10.1155/2013/596517

**Published:** 2013-06-17

**Authors:** Ruken Yuksekkaya, Ali Ekrem Koner, Fatih Celikyay, Murat Beyhan, Ferdag Almus, Berat Acu

**Affiliations:** ^1^Radiology Department, Gaziosmanpasa University School of Medicine, 60100 Tokat, Turkey; ^2^Cardiovascular Surgery Department, Gaziosmanpasa University School of Medicine, 60100 Tokat, Turkey

## Abstract

Chronic-contained aortic aneurysm rupture with vertebral erosion is a rare entity with fatal complications. Multidetector computed tomography (CT) angiography is an important diagnostic method for the evaluation of the aortic aneurysms, their complications, and also the relationship between aneurysm and branching vessels and adjacent structures. We present the multidetector CT angiography findings of a 62-year-old patient with chronic-contained thoracoabdominal aortic aneurysm rupture causing severe vertebral body erosion.

## 1. Introduction

Erosion of the vertebral body caused by an aortic aneurysm is a rare condition. Vertebral erosion secondary to aortic aneurysm may be due to inflammation, infection, Behçet's disease, and tuberculosis [1–3]. It is uncommon in primary aortic aneurysm. In the literature, there are a few case reports about chronic ruptured aortic aneurysms with vertebral body erosion [2–10]. We herein present the multidetector computed tomography (CT) angiography findings of a 62-year-old patient with thoracoabdominal aortic aneurysm causing severe vertebral body erosion. The diagnostic importance of multidetector CT angiography is emphasized in this case report because this condition, in addition to being rare, may have important complications and even can be fatal [3].

## 2. Case Report

**Figure 1:**
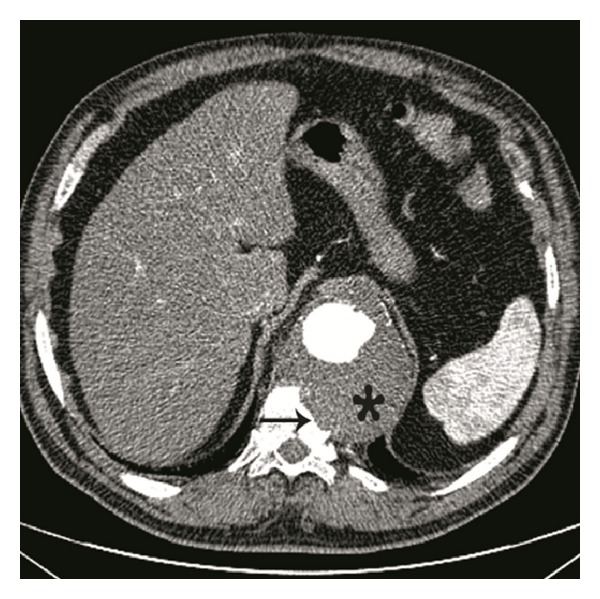
Contrast-enhanced axial multidetector CT angiography image obtained at the level of thoracal 11th vertebra reveals chronic-contained aneurysm rupture of the aorta (*) with vertebral erosion and lysis. Draped aorta sign is present (arrow).

**Figure 2:**
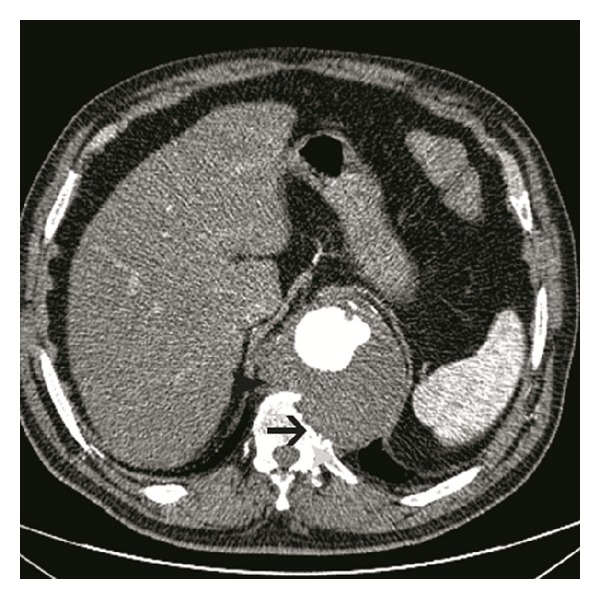
Contrast-enhanced axial multidetector CT angiography image obtained at the level of thoracal 11th vertebra reveals chronic-contained aneurysm rupture of the aorta with vertebral erosion and lysis. Draped aorta sign is present (arrow). Aortic lumen was irregular at the left side.

**Figure 3:**
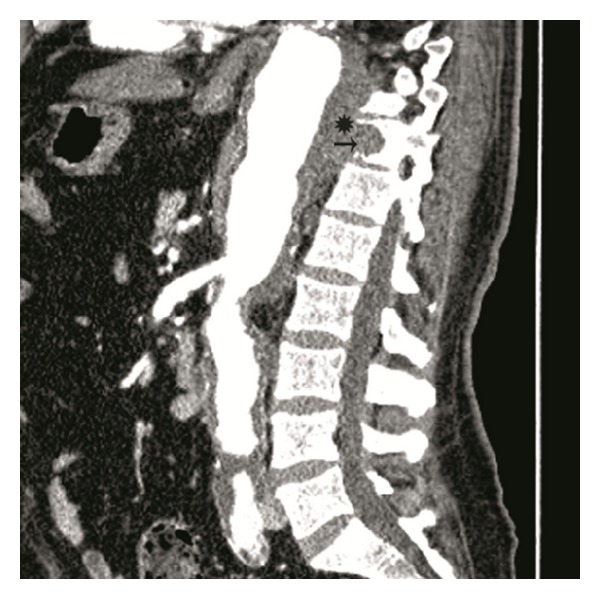
Contrast-enhanced multidetector CT angiography lateral MPR image shows chronic-contained aneurysm rupture (*) of the aorta with vertebral erosion and lysis (arrow).

A 62-year-old man was admitted to our hospital with cough and chest pain. He had a history of treated larynx carcinoma two years ago and a diagnosis of aortic aneurysm. On physical examination, an umbilical hernia, abdominal distension, and a systolic murmur at the auscultation on the abdomen were found. Blood examinations did not reveal any abnormality. Multidetector CT angiography scans were obtained in dorsal decubitus position, during maximum inspiration, by using 8-channel multidetector CT system (GE Healthcare, Milwaukee, WI, USA). Contiguous axial slices with contrast-enhanced CT scans were obtained at 2.5 mm intervals, 0.875 mm slice thickness, and 105 Kvp, 305 mA. All images were obtained at window levels appropriate for mediastinum (window width: 250–400 HU; window level: 40–50 HU). Images were reconstructed with high-resolution algorithm. Multiplanar reformatted (MPR) images were interpreted in various planes. Multidetector CT angiography revealed a thoracoabdominal aortic aneurysm. The maximum diameter of the aneurysmal sac excluding the contained rupture was about 6 cm with a mural thrombus of about 4 cm thickness at about the 12th thoracal vertebral level. There was bone lysis and destruction at the left anterolateral aspect of the 11th vertebral body. The erosion encompassed two-thirds of the vertebral body, and there was no involvement of the spinal canal. The margins of the vertebral erosion were sclerotic and not well corticated. On some images, the posterior wall of the aorta was not completely visualised and could not be seen separately from vertebral body (Figures 1–3). Also, the rim of calcification was not continuous. No hemorrhage was detected. Draped aorta sign was positive (Figures 1 and 2). Multidetector CT angiography findings were interpreted as chronic-contained rupture of the aorta. Lysis and erosion of the vertebral body was attributed to the pressure effect of the pulsatile aortic aneurysm. Because of the poor medical condition of the patient, followup and supportive treatment were recommended without surgical treatment.

## 3. Discussion

Aortic aneurysms are not rare in daily clinical and radiological practice. But they are occasionally accompanied by vertebral erosion and lysis. In our case, chronic-contained rupture and chronic pressure of a primary pulsatile thoracoabdominal aortic aneurysm caused vertebral lysis and erosion. Erosion of the vertebral bodies by an abdominal aortic aneurysm was seen in only 7% of the cases [4]. Chronic-contained aortic aneurysm rupture, also known as sealed or contained leak, was found in only 1.5–3% of abdominal aortic aneurysm [5, 6]. They were associated with retroperitoneal hemorrhage [7]. Erosion into the vertebral colon was a very rare condition in these cases [8–12]. Chronic rupture may be associated with the small tears which can be tamponaded by the vertebral colon [13]. It has been suggested that, when the diameter of aneurysm is small, the aortic wall is relatively strong and a small tear can be limited [5, 13]. Erosion of the vertebra may be caused by the combination of the direct contact of the blood with bone and the repetitive compression by the pulsatile mass of the aneurysm [5, 12]. Also, chronic small leakages might be resulted from inflammation. But contrast enhancement on CT suggesting chronic inflammation was not reported in the literature, as in our case [8–13].

Also, in the literature, the cases with chronic-contained rupture of aortic aneurysm were in general stable clinically and hemodynamically and diagnosed incidentally [13]. Our case was also hemodynamically stable. In addition to the above mentioned mechanisms, normal heart rates and blood pressures may be responsible for the limiting of the small tears of the vessel wall and chronic leakage. 

Aortic aneurysm may cause pain according to the degree of the destruction or neurological deficit caused by the pressure on neural structures [8, 9]. In our case, neurological evaluation was normal. Paraparesis [10], paraplegia [11], low back pain [14], and lumbar hernia [15] were reported in the literature.

Vertebral erosion accompanying aneurysm rupture is a diagnostic challenge. In differential diagnosis, primary or metastatic vertebra tumors, vertebral fractures, osteoporosis, spondylodiscitis or spondylitis, retroperitoneal abscess, Pott's disease, and retroperitoneal tumors should be considered. Diagnosis of the aneurysmal pressure as the cause of the bone erosion is very important and vital to avoid unnecessary diagnostic and treatment options. Moreover, vertebral erosion and aortic aneurysm may develop independently in some infective and inflammatory diseases such as Behçet's disease and syphilitic aortitis. Multidetector CT angiography is a useful diagnostic method in the evaluation of this rare and important condition with possible complications of hemorrhage, paraplegia, and death. It is the most popular diagnostic method for evaluating an aortic aneurysm and provides a detailed analysis of the aortic wall. It is not only useful for the imaging of the aorta but also for the relationships with its branches and adjacent structures such as vertebrae, psoas muscle, and retroperitoneum. By electrocardiography-gating and high spatial resolution, intrathoracic aorta can be easily evaluated without artefacts [16]. Transarterial angiography may be helpful in the detection of the aortic aneurysm but can reveal only luminal pathology without providing any information about mural thrombus, aortic wall, and adjacent structures. Multiplanar reformatted and 3D images may be helpful for the detailed evaluation of the aneurysm in various planes. These advantages of the multidetector CT angiography with MPR and 3D images are also useful during the diagnosis and the management of the chronic-contained aneurysm ruptures. 

The characteristic features of the chronic-contained aneurysm rupture on multidetector CT are draping of the aorta, vertebral erosion, discontinuity of the rim of calcification in the true aneurysm wall, well-defined soft tissue density adjacent to the aorta, psoas muscle involvement, displaced abdominal structures, and no appearance of contrast material in the hematoma, as discussed in the literature [6, 13, 17]. Draped aorta sign is defined as an area in which the posterior aortic wall is unidentifiable as a distinct line from adjacent structures, and the posterior aorta is in close contact with the spine and follows the contours of the vertebrae on one or both sides [17]. Halliday and Al-Kutoubi [17] were the first to describe the “draped aorta” on CT. It is important in differentiating chronic-contained aneurysm rupture from frank rupture and is also helpful in ruling out extra-aortic pathologies to avoid invasive procedures such as biopsy. Besides, the vertebral erosion is also an important CT feature because it takes place at the pathogenesis of the chronic-contained rupture. It is rarely found in frank ruptures [12]. Apter et al. [14] reviewed six patients with chronic-contained aortic aneurysm rupture. In their study group, all cases had draped aorta sign and vertebral erosion. Discontinuity of the rim of calcification in the aneurysm wall, well-defined soft tissue density adjacent to the aorta, psoas muscle involvement, and displaced abdominal structures are important diagnostic CT features, but they may be present in frank ruptures too [6, 18]. Extravasation of contrast material from aorta is diagnostic for frank ruptures. In chronic-contained aneurysm ruptures, no extravasation is observed [6]. Our case matches the above mentioned criteria for chronic-contained rupture.

In conclusion, multidetector CT angiography is the most preferred, noninvasive, accurate, and easy imaging technique in the evaluation of aorta and its branches or adjacent structures. It is well recognized that multidetector CT allows the evaluation of presence, size, and localization of the aneurysms and the possible complications. The awareness and the recognition of typical multidetector CT angiography features of chronic-contained aneurysm rupture for a radiologist is crucial because of the fatal complications of this condition. 

## References

[1] Macedo TA, Stanson AW, Oderich GS, Johnson CM, Panneton JM, Tie ML (2004). Infected aortic aneurysms: imaging findings. *Radiology*.

[2] El Maghraoui A, Tabache F, El Khattabi A (2001). Abdominal aortic aneurysm with lumbar vertebral erosion in Behçet’s disease revealed by low back pain: a case report and review of the literature. *Rheumatology*.

[3] Takahashi Y, Sasaki Y, Shibata T, Suehiro S (2007). Descending thoracic aortic aneurysm complicated with severe vertebral erosion. *European Journal of Cardio-Thoracic Surgery*.

[4] Aydogan M, Karatoprak O, Mirzanli C, Ozturk C, Tezer M, Hamzaoglu A (2008). Severe erosion of lumbar vertebral body because of a chronic ruptured abdominal aortic aneurysm. *Spine Journal*.

[5] Jang JH, Kim HS, Kim SW (2008). Severe vertebral erosion by huge symptomatic pulsating aortic aneurysm. *Journal of Korean Neurosurgical Society*.

[6] Latif S, Wasti A, Grundy DJ, Isdale A, Iveson JMI (1995). Direct erosion of lumbar spine by an abdominal aortic aneurysm, resulting in paraparesis: unusual presentation. Case report. *Paraplegia*.

[7] Mii S, Mori A, Yamaoka T, Sakata H (1999). Penetration by a huge abdominal aortic aneurysm into the lumbar vertebrae: report of a case. *Surgery Today*.

[8] Pereda D, Uriarte C, Barriuso C, Mestres C-A (2005). Vertebral erosion and paraplegia due to expanding thoracic aneurysm. *European Journal of Cardio-thoracic Surgery*.

[9] Kapoor V, Kanal E, Fukui MB (2001). Vertebral mass resulting from a chronic-contained rupture of an abdominal aortic aneurysm repair graft. *American Journal of Neuroradiology*.

[10] Choplin RH, Karstaedt N, Wolfman NT (1982). Ruptured abdominal aortic aneurysm simulating pyogenic vertebral spondylitis. *American Journal of Roentgenology*.

[11] Sterpetti AV, Blair EA, Schultz RD, Feldhaus RJ, Cisternino S, Chasan P (1990). Sealed rupture of abdominal aortic aneurysms. *Journal of Vascular Surgery*.

[12] Ando M, Igari T, Yokoyama H, Satokawa H (2003). CT features of chronic contained rupture of an abdominal aortic aneurysm. *Annals of Thoracic and Cardiovascular Surgery*.

[13] Jones CS, Reilly MK, Dalsing MC, Glover JL (1986). Chronic contained rupture of abdominal aortic aneurysms. *Archives of Surgery*.

[14] Apter S, Rimon U, Konen E (2010). Sealed rupture of abdominal aortic aneurysms: CT features in 6 patients and a review of the literature. *Abdominal Imaging*.

[15] Dobbeleir J, Fourneau I, Maleux G, Daenens K, Vandekerkhof J, Nevelsteen A (2007). Chronic contained rupture of an abdominal aortic aneurysm presenting as a grynfeltt lumbar hernia. A case report. *Acta Chirurgica Belgica*.

[16] Agarwal PP, Chughtai A, Matzinger FRK, Kazerooni EA (2009). Multidetector CT of thoracic aortic aneurysms. *Radiographics*.

[17] Halliday KE, Al-Kutoubi A (1996). Draped aorta: CT sign of contained leak of aortic aneurysms. *Radiology*.

[18] Yu T, Zhu X, Tang L, Wang D, Saad N (2007). Review of CT angiography of aorta. *Radiologic Clinics of North America*.

